# Enoxaparin‐induced bullous hemorrhagic dermatosis at a distant location from the injection site

**DOI:** 10.1002/ccr3.5014

**Published:** 2021-10-28

**Authors:** Abdulrahman F. Al‐Mashdali, Mohamed A. Yassin

**Affiliations:** ^1^ Department of Internal Medicine Hamad Medical Corporation Doha Qatar; ^2^ National Center for Cancer Care and Research Department of Oncology, Hematology and BMT Section Hamad Medical Corporation Doha Qatar

**Keywords:** drug reaction, enoxaparin, hemorrhagic dermatosis, heparin

## Abstract

Heparin‐induced bullous hemorrhagic dermatosis is a benign and self‐limited condition. However, it might cause significant patient frustration. Clinicians should be aware of this rare adverse reaction.

## Graphical abstract

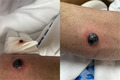

## 1 CLINICAL IMAGE

Heparin‐induced bullous hemorrhagic dermatosis is an infrequent adverse reaction occurring at a distant location from the injection site. It is more commonly seen with enoxaparin than unfractionated heparin. The exact pathogenesis of this condition is unknown. It is a self‐limited reaction causing no significant complication.

**FIGURE 1:**
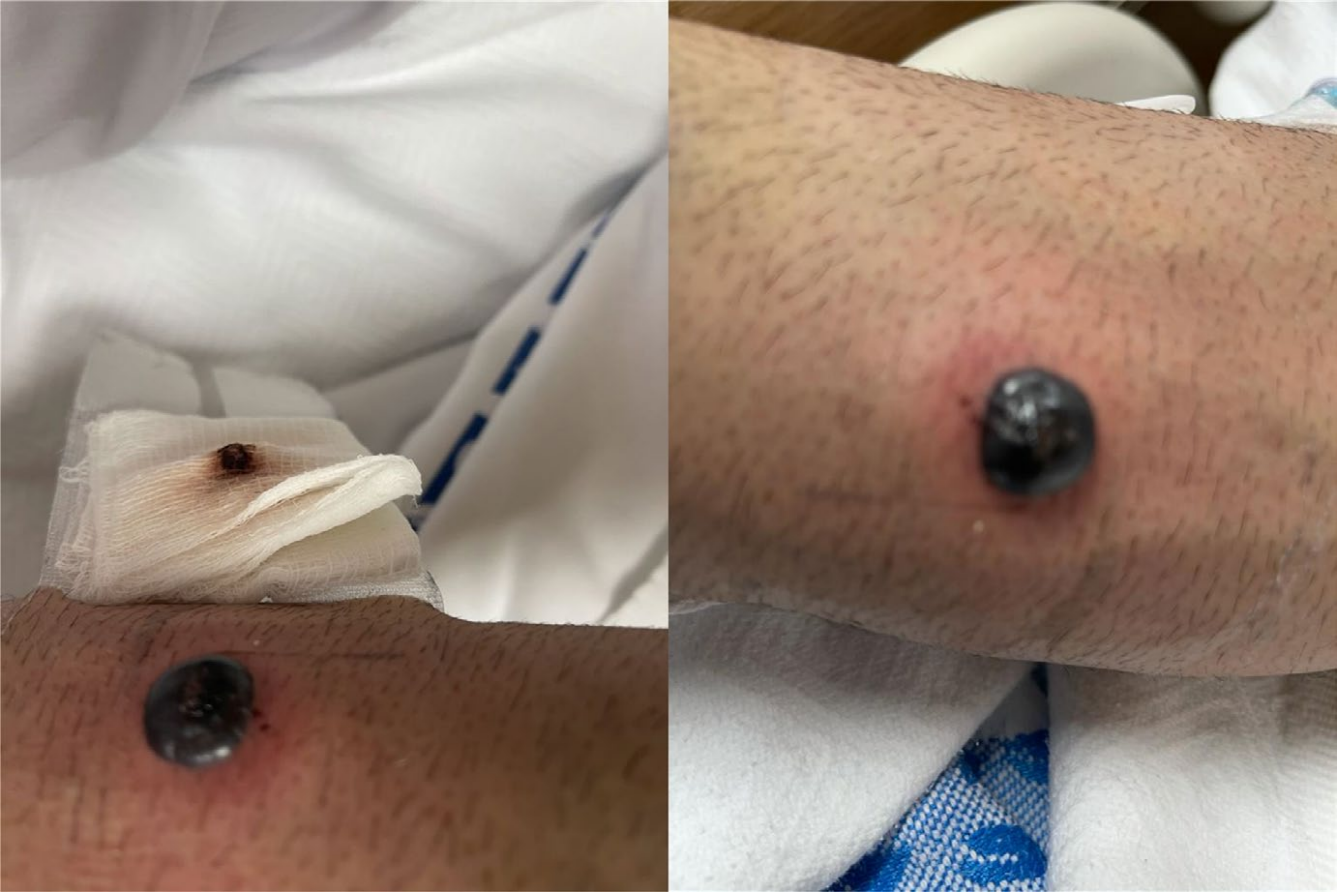
Tense hemorrhagic bullous in the right forearm measuring approximately 3 × 4 cm with surrounding skin erythema (enoxaparin was not injected in this area before)

A 53‐year‐old male with no past medical history presented to our emergency department with severe chest pain. Electrocardiography (ECG) revealed multiple ST segments depression and elevated serum troponin levels. The diagnosis of non‐ST segment elevation myocardial infarction (NSTEMI) was made. Coronary angiography (CAG) showed triple vessel disease. He was started on aspirin, clopidogrel, and therapeutic enoxaparin (90 mg twice daily, in the subcutaneous tissue of the abdomen). He developed an itchy rash in his right forearm on day seven and then progressed into a single tense hemorrhagic bullous (Figure 1). He denied any trauma or receiving any injection in the right forearm. Incision of the bullous was done for symptomatic treatment.

Bullous hemorrhagic dermatosis induced by heparin might be either generalized or localized. It is more prevalent with enoxaparin than unfractionated heparin and usually develops within 5–21 days of heparin initiation. The pathogenesis remains unclear, but it has been proposed that delayed hypersensitivity and acute allergic reactions might involve. Lesions often regress within 7–14 days, and heparin products discontinuation should be decided based on the clinical indication and severity of the lesions. In some cases, an oral anticoagulant is an alternative option for heparin.^1^, ^2^

## CONFLICT OF INTEREST

The authors have no conflict of interest to declare.

## AUTHOR CONTRIBUTIONS

AFA contributed to data collection, literature review, consent taking, and manuscript writing. MAY contributed to the final manuscript review and editing. All authors reviewed and approved the final version of the article.

## CONSENT

Written informed consent was obtained from the patient for the publication and the accompanying images of this case report.

## Data Availability

Available upon request.
